# Exploring the Antibiotic Resistance Burden in Livestock, Livestock Handlers and Their Non-Livestock Handling Contacts: A One Health Perspective

**DOI:** 10.3389/fmicb.2021.651461

**Published:** 2021-04-20

**Authors:** Rachel A. Hickman, Thongpan Leangapichart, Kamonwan Lunha, Jatesada Jiwakanon, Sunpetch Angkititrakul, Ulf Magnusson, Marianne Sunde, Josef D. Järhult

**Affiliations:** ^1^Department of Medical Biochemistry and Microbiology, Zoonosis Science Center, Uppsala University, Uppsala, Sweden; ^2^Section for Animal Health and Food Safety, Norwegian Veterinary Institute, Oslo, Norway; ^3^Department of Clinical Sciences, Swedish University of Agricultural Sciences, Uppsala, Sweden; ^4^Research Group for Animal Health Technology, Faculty of Veterinary Medicine, Khon Kaen University, Khon Kaen, Thailand; ^5^Department of Medical Sciences, Zoonosis Science Center, Uppsala University, Uppsala, Sweden

**Keywords:** one-health approach, antibiotic resistance, zoonotic transmission, *E. coli*, livestock, pigs, meat-production

## Abstract

Antibiotics are freqeuently used in the livestock sector in low- and middle-income countries for treatment, prophylaxis, and growth promotion. However, there is limited information into the zoonotic prevalence and dissemination patterns of antimicrobial resistance (AMR) within these environments. In this study we used pig farming in Thailand as a model to explore AMR; 156 pig farms were included, comprising of small-sized (<50 sows) and medium-sized (≥100 sows) farms, where bacterial isolates were selectively cultured from animal rectal and human fecal samples. Bacterial isolates were subjected to antimicrobial susceptibility testing (AST), and whole-genome sequencing. Our results indicate extensive zoonotic sharing of antibiotic resistance genes (ARGs) by horizontal gene transfer. Resistance to multiple antibiotics was observed with higher prevalence in medium-scale farms. Zoonotic transmission of colistin resistance in small-scale farms had a dissemination gradient from pigs to handlers to non-livestock contacts. We highly recommend reducing the antimicrobial use in animals’ feeds and medications, especially the last resort drug colistin.

## Introduction

Understanding zoonotic interactions is critical in controlling the plethora of infectious agents that affect our health as highlighted by COVID-19. One important zoonotic concern is the global challenge of antimicrobial resistance (AMR), which has been exacerbated by the overuse and misuse of antibiotic drugs both by the implementation in humans and domestic animals. It has been estimated that 60% of human pathogens are acquired from other animal species but quantification of transfer from humans to animals has been understudied and remain relatively unknown (Sheppard et al., 2018). Therefore, we used Thailand as a model country to explore zoonotic AMR patterns in the commensal bacteria *Escherichia coli* (*E. coli*), isolated from livestock pigs (P), contact handlers (C), and non-livestock contact individuals (NC) in the farm’s proximity. To understand which antibiotic drugs pose AMR risks and assess zoonotic AMR transfer events, we utilized antimicrobial susceptibility testing (AST) and whole genome sequencing (WGS) methods in a One Health multi-factorial model consisting of different meat production intensities and subjects within the farm environment.

Traditional antimicrobial usage (AMU) in the livestock sector aims to treat bacterial infections, provide prophylactic treatment to prevent infectious manifestations (McEwen and Fedorka−Cray, 2002; Lugsomya et al., 2018; McEwen and Collignon, 2018), and as growth promoters in animal feed to help generate higher yields (Hughes and Heritage, 2004; Van Boeckel et al., 2015). This paradigm is still followed in low- to middle-income countries (LMICs); whilst there has been a shift in many high-income countries where AMU is now banned for growth promotion and limited to treatment of sick animals with veterinary prescription (Call to phase out prophylactic use of antimicrobials in livestock; No Author, 2011) and reinforced by different interventions depending on the country (Carmo et al., 2018). Unfortunately, in LMICs regulatory framework is often missing or compliance to existing ones is weak (Sommanustweechai et al., 2018), in addition there is often limited surveillance or data on AMU (Carrique-Mas et al., 2020), and further complicated by many livestock producers understanding little about the agents they are using, and further exacerbated by a lack of knowledge in AMR (Ström et al., 2018).

Thailand is a middle-income South-East Asian country and popular travel destination with foreign tourists (Tängdén et al., 2010; Record 38.27m tourists in 2018; 41m expected in 2019, 2019) where antibiotics are freely available over-the-counter with a few exceptions (Sommanustweechai et al., 2018). In locations where there is high AMU, there is also a high AMR burden (O’neill, 2016). This is of critical concern with antibiotic drugs, such as colistin, that are used as a last-resort drugs in humans to treat extensively resistant bacterial infections, e.g., carbapenem resistant Enterobacteriaceae (Wangchinda et al., 2018; Prakobsrikul et al., 2019) where very limited treatment options are available. Where last-resort drugs, such as colistin, are frequently used in livestock and in clinical settings generalized AMR can occur making these crucial drugs void and some bacterial infections untreatable (McEwen and Collignon, 2018). A previous Thai study found a high AMR prevalence in *E. coli* from chicken and pig farms; isolates were resistant to several antibiotics: ampicillin (97.8 and 94.4% for chickens and pigs, respectively), ciprofloxacin (73.3 and 21.1%), gentamicin (42.2 and 35.6%), and colistin (22.2 and 24.4%)(Nguyen et al., 2016). Whilst a study from the neighboring country Cambodia, saw an Extended spectrum-beta lactamase (ESBL) *E. coli* prevalence of 20% in humans and 23% in livestock (Atterby et al., 2019).

We wanted to implement the principles of One Health by performing a muliti-displinary study in a LMIC model country where AMR knowledge is often scarce due to limited resources and the few studies published are often limited in scope for potential risk factors that may be pivotal in reducing morbidity and mortality. Therefore, we used as Thailand is a LMIC livestock model to provide much needed evidence-based recommendations on AMU in LMICs in order to save the clinical effect of important antibiotics.

## Materials and Methods

### Study Area, Farms, Participants, and Fecal Sample Collection

This study was conducted in the Khon Kaen province of North-East Thailand during September-December 2018 in 166 pig farms. Farms were separated on production scale, all farms in this study were considered either small- or medium-scale farms. In this study small-scale farms were defined as having ≤50 sows (often family owned), whilst medium-scale farms were defined as having ≥100 and ≤500 sows and (company owned). In total there was 115 small-scale and 51 medium-scale farms. At each farm we tried to obtain a fecal samples from a contact human and a non-contact human and up-to 10 pigs (depending on the number of pigs on the farm) that were pooled together. Overall we collect 143 pig, 90 contact human and 54 non-contact human fecal sample and stored in accordance to the methods described by Lunha et al. (2020) at the Faculty of veterinary medicine, Khon Kaen University.

### Bacterial Isolation on Selective Media and Antimicrobial Susceptible Testing

Pig rectal swabs from the same farm (*n* ≤ 10) were pooled in 25 mL of buffered peptone water for enrichment, information regarding numbers of pooled pig rectal swabs from specific farms is provided in Lunha et al. (2020). One gram of human faces was enriched in 5 mL of buffered peptone water. After incubation at 37°C overnight, 10 μL of suspension was plated onto MacConkey agar (Oxoid, Hampshire, England) containing cefotaxime 1 μg/mL (Sigma-Aldrich), CHROMagar^TM^ mSuperCARBA^TM^, and CHROMagar^TM^ COL-APSE (CHROMagar, Paris, France). Different morphologic types of colonies were collected and identified by matrix-assisted laser desorption ionization time of flight (MALDI-TOF, Microflex, Bruker Daltonik GmbH). All isolates were re-streaked from frozen stocks onto blood-agar plates, incubated at 37°C 18 h and checked for potential contamination before AST. All AST were performed using the Sensititre EUVSEC AST microdilution plates using Sensititre Mueller Hinton Broth tubes and the Sensititre AIM pipetting robot (Thermo Fisher Scientific, Fair Lawn, NJ, United States). The AST was done in accordance to the Sensititre EU Surveillance Salmonella/E. coli EUVSEC Plate workflow (Thermo Scientific Sensititre Plate Guide for Antimicrobial Susceptibility Testing, 2018). Quality control was performed with *E. coli* ATCC 25922 isolate and produced the expected results. All plates were manually read with the sensititre manual viewbox and photographed for documentation. Isolates were catogorized in accordance to definitions of Enterobacteriaceae in Magiorakos et al. (2012) where multi-drug resistance (MDR) was defined in as when isolate is non-susceptible to at least 1 agent in ≥ 3 antimicrobial categories, extensively multi-drug resistance (XDR) when the isolate is non-susceptible to at least 1 agent in all but 2 or fewer antimicrobial drug catergories and pan mutli-drug resistance when the isolate is non-susceptible all antimicrobial drug catergories.

### DNA Extraction and Whole Genome Sequencing

All isolates were re-streaked from frozen stocks onto blood-agar plates, incubated at 37°C 18 h and checked for potential contamination before DNA extraction. DNA was extracted using 10–15 μL of a fresh bacterial colonies and processed using the Qiagen DNeasy blood and tissue kit in accordance to the manufacturer’s instruction (Qiagen, Hilden, Germany). All processed samples had a minimum DNA concentration of 30 ng/μL verified using the dsDNA HS Assay kits on a Qubit Fluorometer (Thermo Fisher Scientific, Fair Lawn, NJ, United States) and OD260/280 in the range of 1.8–2.0 verified by the NanoDrop spectrophotometer (Thermo Fisher Scientific, Fair Lawn (NJ), United States). Samples were shipped on dry ice to the Novogene sequencing facility (Novogene, Hong Kong, China) and sequenced on the Novoseq Illumina platform (Illumina, San Diego, CA, United States) and produced ∼5 GB of 150 bp pair-end sequencing reads per isolate.

### Phenotypic and Genomic Data Analysis

**FIGURE 1:**
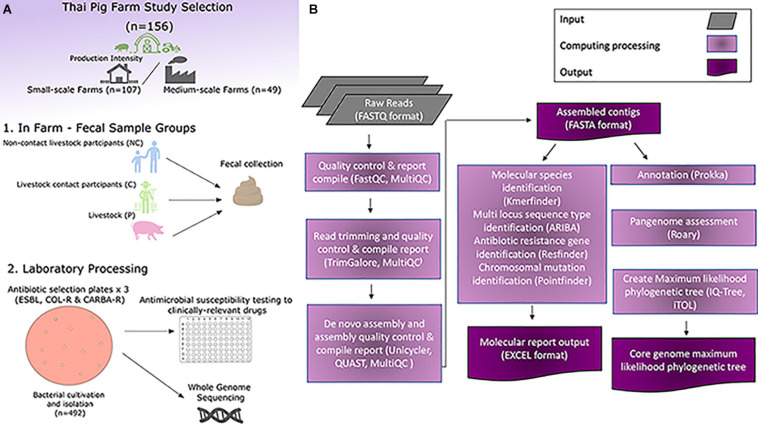
Overview of experimental and computational set-up of the study. **(A)** Experimental workflow of the study. **(B)** Computational analysis work-flow of the study.

All AST data was converted into either a susceptible, intermediate or resistant classification for each isolate and all the individual tested antibiotic drugs in accordance to EUCAST clinical breakpoints (European Committee on Antimicrobial Susceptibility Testing, 2013). Then further processed in Python (3.8.3rc1 Documentation, 2020) using the matplotlib, pandas and seaborn packages. Genomic data processing was performed on the high computing capacity provided by SNIC through Uppsala Multidisciplinary Centre for Advance Computational Science (UPPMAX). All processing of the whole genome sequences was done with open software with an in-house bioinformatics pipeline. Our pipeline consists of four main modules: the first module performed a quality control (QC) assessment of the raw sequence files and trimming the sequence reads with FastQC (Wingett and Andrews, 2018), MultiQC (Ewels et al., 2016), and Trim Galore (Babraham Bioinformatics, 2020); the second module performed *de novo* assembly with Unicycler with minimum contigs of 400 bp produced (Wick et al., 2017) and assembly QC was performed by QUAST and MultiQC (Gurevich et al., 2013; Ewels et al., 2016); the third module compiled a molecular output excel report from running our genomic data through KmerFinder (Larsen et al., 2014), ARIBA (Hunt et al., 2017), ResFinder (Zankari et al., 2012), and PointFinder (Zankari et al., 2017); and the fourth module produced a core genome maximum likelihood phylogenetic tree by running Prokka (Seemann, 2014) to annotate all the isolate genomes, Roary (Page et al., 2015) to assess the pangenome and generate the core genome alignment, and IQ-Tree2 (Quang et al., 2020) to generate maximum likelihood phylogenetic tree (Figure 1). Downstream genomic data processing was done in Python (3.8.3rc1 Documentation, 2020) using the matplotlib, pandas and seaborn packages. Visualization of the maximum likelihood phylogenetic tree was done using iTOL webtool (Letunic and Bork, 2019). Additional SNP detection of matching ST isolates between groups on the same farm was done using snippy (Seemann, 2015), detection of virulence genes was done using the virulencefinder pipeline (Joensen et al., 2014) and pangenome query difference tables were produced between human and pig groups using Roary query pangenome function (Page et al., 2015).

### Ethical Approval

This study was performed in accordance to the Helsinki declaration for the human subjects and the EU Directive 2010/63/EU for animal experiments; the protocol involving human participants and animals was approved by the Khon Kaen University Ethics Committee (Project ID: HE612268 and 0514.1.75/66, respectively). Informed consent for each human subject was obtained after an explanation of the experimental procedures. Pig samples were collected at the farm with the farm owners permission of the pig herd.

### Data Availability

Raw sequence data can be obtained from the European Nucleotide Archive (ENA) under the project accession number PRJEB38313. All sequence data from computation workflow is compiled in Supplementary File 1, virulencefinder unique results in Supplementary File 2 and genomic difference output is provided in Supplementary File 3. All scripts and Python code and metadata can be provided on request.

## Results

### Selection Criteria of Bacterial Isolates for Phenotypic and Genomic Analysis

To explore zoonotic AMR patterns in *E. coli* isolates we examined 156 farms in Khon Kaen province of North-East Thailand, utilizing our livestock and human participant One Health study model. A total of 143 pooled pig samples, 90 C and 54 NC human fecal samples and were collected and processed. We generated an isolate collection of 492 *E. coli* isolates derived from culturing fecal material on agar plates selective for ESBL-producing, carbapenemase-producing, or colistin-resistant gram-negative bacteria. From the 107 small-scale farms (SSF) 174 isolates were from livestock pigs (P), 99 isolates from livestock producers (C), and 57 isolates were from and non-livestock contact individuals (NC). From the 49 medium-scale farms (MSF), 94 isolates were from Ps, 44 isolates from Cs, and 24 isolates were from NCs. Several studies in farms in South-East Asia have looked at *E. coli* isolates from different livestock and farm workers (Nguyen et al., 2016; Lugsomya et al., 2018; Prakobsrikul et al., 2019); to build upon these studies we wanted to additionally assess farm production size in relation to P, C, and NC and look for differences between the six groups. To record significant differences between the isolate groups we used AST and WGS with *in silico* methods (Figure 1) to explore the genomic diversity (Read and Massey, 2014; Abdelgader et al., 2018; Boehmer et al., 2018).

### AMR Is Prolific With Average AST Results Far Above Clinical Breakpoints

**FIGURE 2:**
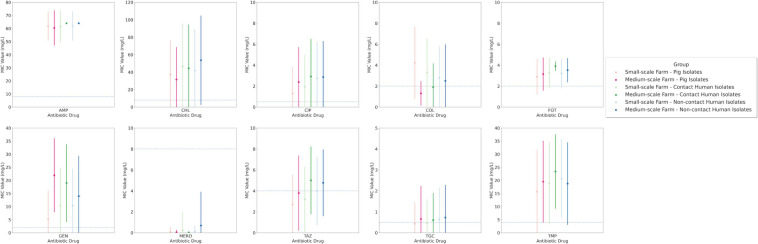
Average Minimal inhibitory concentration (MIC) for drug investigation group to each drug reagent with their respective standard deviation and clinical breakpoints for each drug are displayed across the *y*-axis.

Utilizing the *E. coli is*olates to assess AMR, we could establish AMR patterns to demonstrate single antibiotic drug efficacy. Several antibiotic drugs had an average group MIC concentration that exceeded the clinical breakpoint: ampicillin, chloramphenicol, ciprofloxacin, cefotaxime, gentamicin, and trimethoprim (Figure 2). This was not surprising for ampicillin or cefotaxime due to the selective culturing method, enriching, e.g., for ESBL *E. coli*. Whilst for other drugs we observed clustering around the clinical breakpoints, e.g., colistin, ceftazidime, and tigecycline. Yet again these results were not surprising for colistin or ceftazidime due to the selection for ESBL and colistin-resistant *E. coli.* Our results demonstrate that ceftazidime and tigecycline both had a higher phenotypic AMR bias in the human isolates especially those from MSFs. This was not the case for colistin where there was a resistance bias toward SSFs, with the highest results occurring in the SSF-P isolates with an average MIC concentration of 4.2 mg/L, second highest in SSF-C isolates with an average MIC concentration of 3.3 mg/L, and third was the SSF-NC isolates with an average MIC concentration of 2.8 mg/L. These results suggest SSF pigs as a colistin AMR reservoir and zoonotic transfer to the human population. Colistin resistance in Thailand has been frequently reported (Nguyen et al., 2016; McEwen and Collignon, 2018; Ström et al., 2018; Wangchinda et al., 2018; Collignon and McEwen, 2019) but here we report an accumulation in SSFs and signs of zoonotic transmission to humans. However, for chloramphenicol and cefotaxime we saw a preference for human-associated host AMR with a bias toward MSFs rather than SSFs. The average MIC value for chloramphenicol had the highest average MIC value in MSFs in NCs 53.7 mg/L, followed by 44.4 mg/L in Cs and lowest in Ps 31.7 mg/L. Cefotaxime had the highest average MIC value in MSF in C 3.9 mg/L, followed 3.5 mg/L in NCs and lowest in Ps 3.1 mg/L. The only drug that had wide-scale average MIC concentration that indicated susceptibility in all groups was meropenem. In our isolate collection only 6 isolates were cultivated from our carbapenem-selective plates, out of these isolates only 2 were confirmed by AST testing to be meropenem resistant (Supplementary Figure 1). Both isolates were obtained from humans on independent farms; one from a NC and the other from a C. To ensure our findings were statistically relevant we performed one-way ANOVA statistical tests with a Bartlett’s test for each antibiotic based on the group MIC values to see significant differences both in the mean and the standard deviation. Four antibiotic drugs had a significant difference between groups: chloramphenicol, colistin, cefotaxime, and meropenem. In addition we also converted our group isolates into the susceptible (S), intermediate (I) or resistant (R) categories using the EUCAST breakpoints (Supplementary Figure 2). In this analysis we observed the same trends for the four antibiotic drugs that had significant differences in the MIC-value comparison. For instance we still observed with colisin the zoonotic spill-over effect in the SSFs where overall resistant isolates in the groups were 55, 38, and 29% in P, C and NC, respectively, compared to in MSFs 5.3, 13.6, and 20.8% in the same respective order.

We also wanted to examine the collateral damage of AMR of multiple agents in different antibiotic classes in our isolate data. As others have stated that this is a prolific AMR problem in South-East Asia, from our data we can also confirm this problem both in pigs and humans (Lai et al., 2014; Chereau et al., 2017). Our results are summarized in Table 1 showing the distribution of drug resistance from 0 up to 8 different classes of antibiotics. Following the definitions provided by Magiorakos et al. (2012) we found the most extensive multi-drug resistant (XDR) isolates in the NC cohort. We also found more XDR in the MSFs compared to the SSFs in all groups, with the highest occurrence difference being in P and C. Further, we observed significantly more multi-drug resistant (MDR) in the MSF-C group compared to the SSF-C group.

**TABLE 1:** Distribution of isolates based on their resistant drug classes amongst sample groups.

	Isolation sources					
	P		C		NC	
	SSF	MSF	SSF	MSF	SSF	MSF
Antibiotic resistances to 0–2 drug classes	41.1	36.2	31.3	20.5	31.6	33.3
Multi-drug resistant (MDR)	56.9	58.5	65.7	70.5	57.9	54.2
Extensive multi-drug resistant (XDR)	1.7	5.3	3.0	9.1	10.5	12.5

### Diversity of ARGs in Given Niches Allows Commensal Bacterial Sustainability

**FIGURE 3:**
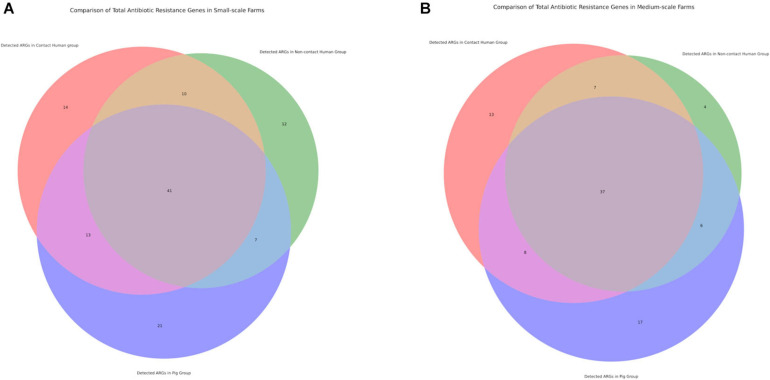
Venn diagrams displaying number of antibiotic resistance genes (ARGs) shared between different investigation groups. **(A)** Comparison of total antibiotic resistance genes in small-scale farms. **(B)** Comparison of total antibiotic resistance genes in medium-scale farms.

**FIGURE 4:**
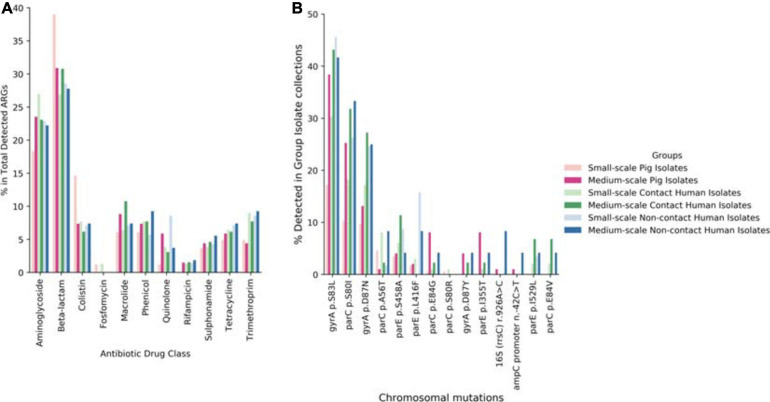
Overview of total ARGs and chromosomal mutations. **(A)** Display of % of total ARGs according to antibiotic drug class for each investigation group of this study. **(B)** Display of % of antibiotic resistant associated chromosomal for each investigation group of this study.

Following our phenotypic data, we continued with genotypic characterization. Unlike other preceding studies we used WGS of our 492 isolates to see the full extent of the antibiotic resistance genes (ARGs) and known chromosomal mutations present. Within our data we saw higher diversity of ARGs in the SSFs compared to the MSFs (Figure 3), consistent with a less regulated and uniform AMU in family owned SSFs as compared to company owned MSFs. The majority of these ARGs were in the aminoglycoside, beta-lactam, colistin, fosfomycin, rifampicin, quinolone and trimethoprim antibiotic drug classes (Figure 4A). A total of 118 different ARGs were discovered in the SSFs compared to 92 ARGs in the MSFs, with 31 unique to SSFs and 6 unique to MSFs (Supplementary Table 1). The most striking difference was observed in the SSF-P group where resistance genes to beta-lactam and colistin antibiotic drug classes accounted for 39 and 15% of total ARGs, respectively. The abundance of colistin ARGs in the SSF also correlated with our previously discussed phenotypic results where the highest abundance of colistin resistance genes was observed in Ps followed by Cs and lastly by NCs. We suspect this is due to the fact that it is common for colistin to be used in pig feed and treatments given to SSF-Ps, and consistent with reports of high colistin resistance prevalence in Thai *E. coli* isolates from livestock (Nguyen et al., 2016). We did not detect the same correlation with the beta-lactam ARGs where pigs in SSFs had the highest abundance whilst the other groups appeared to have similar values.

Looking in our WGS dataset we were also interested in detecting chromosomal mutations that confer known antibiotic resistance (Figure 4B). From our literature-supported results, most of the chromosomal mutations conferred resistance to the quinolone antibiotic drug class with the exception of one associated with kasugamycin resistance and the other to ampicillin and clavulanic acid resistance and cephalosporin drugs. We found three point mutations that were present in all sample groups ≥10%, these were: *gyrA* S83L (Everett et al., 1996), *parC* S80I (Kumagai et al., 1996), and *gyrA* D87N (Sáenz et al., 2003). All of these mutations are well-known to confer quinolone resistance and are frequently seen both in human and farm animal samples. All the detected chromosomal mutations in our dataset had a human rather than animal bias and were generally biased toward MSFs compared to SSFs.

### Prevalence of ESBL Genes Was High in All Farms Whilst Colistin Resistance Genes Were Observed in Distinctive Groups

From our genomic analysis we wanted to observe the prevalence of ESBL and colistin resistance genes within our isolate groups (Table 2). We saw a high prevalence of ESBL genes in all groups with the lowest value being 96.6% in MSF-C group, this was much higher than expected due to previous reports in South-East Asia (Nguyen et al., 2016; Atterby et al., 2019). Despite the higher prevalence in our study compared to other reports, the observation of ESBL genes matches our phenotypic results as the average MIC for all groups as was above the clinical breakpoint for cefotaxime, a third-generation cephalosporin (Figure 2). We also saw the same trend for colistin resistance as seen in phenotypic and ARG diversity results. There was a diminishing resistance trend in SSF from P, C to NC, whilst the trend was in the opposite direction on MSF. It should be noted that exact resistance prevalence per farm cannot be directly compared between human and pig samples, as human samples were obtained from single individuals, and pig samples from pools of up to 10 swabs from different pigs. However, the difference in trends is not dependent on exact prevalence, and thus also these results suggest the highest colistin AMU on SSFs, that could be a hot-spot for colistin resistance development and zoonotic spread, unless colistin in pigs is prohibited.

**TABLE 2:** Farm prevalence of ESBL and colistin resistance genes.

	P Isolates group			C Isolates group			NC Isolates group			Summary		
	
Resistance prevalence	SSF	MSF	Total within sample group	SSF	MSF	Total within sample group	SSF	MSF	Total within sample group	Total within small-scale farms	Total within medium-scale farms	Overall total
ESBL	(94/95) 98.9%	(47/48) 97.9%	(141/143) 98.6%	(59/61) 96.7%	(28/29) 96.6%	(87/90) 96.7%	(40/41) 97.6%	(13/13) 100%	(53/54) 98.2%	(106/107) 99.1%	(49/49) 100%	(155/156) 99.4%
Colistin	(59/95) 62.1%	(4/48) 8.3%	(63/143) 44.1%	(25/61) 41.0%	(4/29) 13.8%	(29/90) 32.2%	(11/41) 26.83%	(4/13) 30.8%	(15/54) 27.8%	(66/107) 6 1.7%	(10/49) 20.4%	(76/156) 48.7%
												

### Host-Specific Bacterial Strain Preferences Exist With ARG Acquisition Being Dependent on Farm Type

**FIGURE 5:**
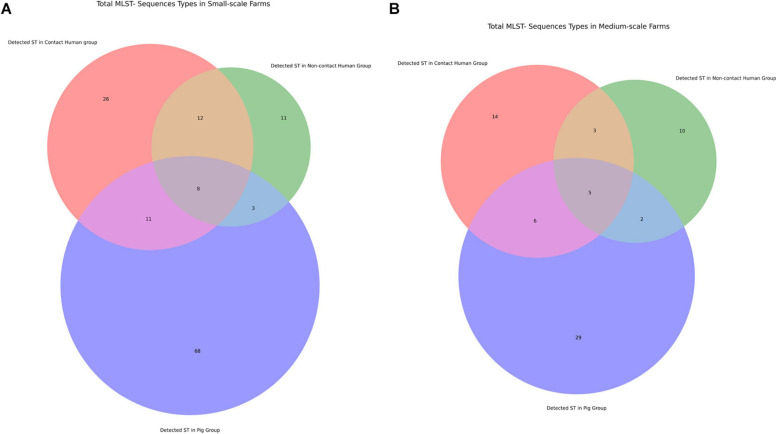
Venn diagram of Multi Locus Sequence Types shared between different investigation groups. **(A)** Comparison of STs in small-scale farms. **(B)** Comparison of total STs genes in medium-scale farms.

To assess the clonality of the isolates from the different study groups we did *in silico* multi-locus sequence typing analysis to identify the sequence types (ST). Then we looked for the most frequent ST in each group (Table 3) and plotted overlapping STs in the different groups (Figure 5). There was a large variation of STs among our isolates and the data suggests some ST types having a high propensity toward a given host (i.e., a large ST overlap between contact and non-contact humans in small-scale farms in Figure 5, and a large number of STs only present in pigs) (Sheppard et al., 2018). However, there were also signs of clonal transfer or shared STs between pigs and humans: (i) ST48 was the most common ST in small-scale farm pigs and contact humans but in no other groups; (ii) the ST overlaps between contact humans and pigs were larger than the overlaps between pigs and non-contact humans in both farm types. To further investigate the clonality of the different isolates within our collection we generated a maximum-likelihood phylogenetic tree (Supplementary Figure 2). From our maximum-likelihood phylogenetic tree we did not observe clonality from farm isolates, we only saw some clustering based on MLST type and sample group. ST48 has been widely reported for harboring ESBLs mainly in LMICs or from imports (Seenama et al., 2019; Tian et al., 2020) but also from human contamination in drinking water in a high-income country (Madec et al., 2016). However, we concluded that several sequence types were circulating within the farm environment with different host preferences that all may play a key role in AMR.

**TABLE 3:** Distribution of MLST sequence types amongst sample groups.

Isolation source	Farm size	Most common ST	Frequency	All STs in group
P	SSF	ST48	17	90
	MSF	ST6449	17	42
C	SSF	ST48	8	57
	MSF	ST10	7	28
NC	SSF	ST1193	6	34
	MSF	ST38 ST216 ST515 ST1193	2	20

To further investigate clonality we examined total variant differences between matching ST isolates in different groups found on the same farm (Table 4). We saw 6 instances where ≤25 variants were detected between group isolates, the majority of these were between C and NC isolates where 3 were from SSFs and 2 from MSFs and one case between a C and P on an MSF. We assumed this bias was due to gene differences required between pigs and humans, such as virulence genes. To confirm this we did virulence gene assessment using virulencefinder (Joensen et al., 2014) but only found four different genes between the human isolates and P isolates, these were *cma*, air and *eilA* in the human sourced isolates and *stb* in Ps (Supplementary File 2). We also use the query pangenome function of the Roary pipeline (Sitto and Battistuzzi, 2020), however we did not see any conclusive differences (Supplementary File 3). We tended to observe higher clonality amongst isolates from the same host and similarity of ARG carriage depending on the farm type, this was most clearly observed between small-scale farms between pigs and contact humans with colistin resistance. From further analysis we were able to detect the origin of replication of multiple plasmids (Supplementary Figure 3), thus supporting that transient zoonotic transmission could allow for acquisition of ARGs by horizontal gene transfer to the host’s endogenous *E. coli* population, as we could observe ST differentiation, but no significant difference in ARG carriage depending on the antibiotic drug. In our data set we only saw one shared strain with 11 SNP or INDEL genomic variants difference between a contact human and a pig on farm 120, a medium-scale farm. We could not see extensive zoonotic sharing of clones between human and pigs. The exception in farm 120 is likely the result of a transient zoonotic transmission event, where the transient bacterial strain could disseminate any of its ARGs to the host’s endogenous Gram-negative bacterial community thus giving rise to new antibiotic resistant strains. The frequency of these events and the amount of antibiotics used both in humans and pigs as a selective pressure for ARGs maintenance will affect the impact.

**TABLE 4:** Variant differences between groups with matching STs.

Group comparisons	Farm size	Farm number	ST	Number of isolates in 1st group	Variant differences in 1st group	Number of isolates in 2nd group	Variant differences in 2nd group	Variant differences between 1st and 2nd group
C compared to NC	SSF	17	10	1	–	1	–	17,796
	MSF	64	515	1	–	2	25	163
	SSF	90	58	1	–	1	–	2,442
	SSF	90	542	1	–	1	–	14
	SSF	98	10,562	2	15	2	21	25
	SSF	103	278	1	–	1	–	14
	MSF	123	58	1	–	1	–	20
	MSF	126	38	1	–	1	–	24
C compared to P	SSF	16	206	1	–	1	–	10,930
	SSF	54	48	1	–	1	–	12,184
	SSF	59	10	1	–	1	–	12,391
	SSF	60	10	1	–	1	–	10,062
	MSF	120	6,786	1	–	1	–	11
	MSF	128	10	1	–	1	–	19,407
NC compared to P	SSF	8	10	1	–	1	–	18,242
	MSF	63	457	1	–	2	0	76,403

## Discussion

From the few previous published studies it has become apparent that there is high AMU burden in South-East Asian LMICs. Additionally in Thailand like several other LMICs antimicrobial pharmaceuticals are generally available over-the-counter, as well as in medicated feed and due to their cost old antibiotics are often recycled for alternative uses (Sommanustweechai et al., 2018). For instance the drug fosfomycin (in addition to beta-lactamase inhibitors and carbapenems) requires a special permit for purchase in Thailand, therefore it would be assumed not to be used in farm animals (Sommanustweechai et al., 2018). Potential explanations for resistance to fosfomycin in livestock still being detected include; use of recycled fosfomycin prescriptions, zoonotic transfer from humans to livestock, or unauthorized sale of fosfomycin as an animal treatment or feed additive. For the other special-permit drugs that belong in the class of carbapenems we saw low AMR prevalence with only two isolates detected in two human individuals (Supplementary Figure 1). We assume this is a result of these drugs being expensive, hard to acquire outside the hospital environment, and potentially that they require intravenous administration. Hopefully inaccessibility in treating animals remains in place to help maintain the susceptibility of these drugs. However, we may see more carbapenem resistance in the pig populations may be seen in the future either from zoonotic transfer events or if an informal market for these drugs occur due to a lack of other antibacterial treatment options.

Utilizing our One Health multi-factorial livestock/human model in Thailand and AST of 492 *E. coli* isolates, we observe prolific AMR to several antibiotic agents especially in MSF where a majority of isolates were MDR or XDR (Table 1). These results corroborate earlier findings from the same study population but using disc diffusion AST analysis of random *E. coli* isolates (not selectively cultured, i.e., not using antibiotic-containing media) (Lunha et al., 2020). In the present study, all isolates were resistant to ampicillin and all groups had an average MIC above the clinical breakpoint for cefotaxime (Figure 2). We also observed an average MIC above the clinical breakpoint for the antibiotics chloramphenicol, gentamicin and trimethroprim/sulfamethoxazole. Additionally we could see indications of zoonotic spill-over of colistin resistance within SSFs with average MIC P > C > NC, i.e., a decreasing colistin MIC gradient.

From WGS analysis we saw high overall farm prevalence of ESBLs detection ranging from 96.6 to 100% across all the investigatory groups, whilst overall farm prevalence of colistin ARGs was 48.7% with the highest group being in the SSF-P with a detection rate of 62.1% (Table 2). The ESBL prevalance was much higher than expected when compared to previous reports in South-East Asia (Nguyen et al., 2016; Atterby et al., 2019). The most common ESBL gene detected was *blaCTX-M-14* accession number AF25622; 43 other ESBL genes were also identified. Whilst the most common colistin resistance gene was *mcr-1.1* accession number KP347127, whereas 12 other colistin resistance genes were also detected in our data set. The colistin ARG detection frequencies had the same zoonotic spill-over indication in SSFs as the AST data (P > C > NC), therefore providing a plausible molecular mechanism for the phenotypic observation seen. From the WGS data we *in silco* analyzed MLST of each isolate; in farms where the same MLST was detected in different sample groups, additional variant analysis was performed. We observed only one case of suspected clonality between human and pig isolates (same ST with less than 25 variants) (Table 2), indicating limited zoonotic sharing of bacterial strains. The most frequent ST shared between P and C in SSFs was ST48, a common *E. coli* ST found in livestock and infamous for ESBL carriage (Seenama et al., 2019; Tian et al., 2020), but no clonal similarity was found when performing the additional variant analysis. Therefore we conclude that horizontal gene transfer is likely the major mechanism of zoonotic ARG dissemination in our study, and potentially in LMIC farm environments in general.

Strengths of this study include providing a large isolate collection that has AST data coupled with extensive molecular data on sequence types, ARGs and clonal similarities between isolates. Furthermore, a One Health, multi-disciplinary approach was used, and concurrent samples from humans and animals were obtained. From our approach we could select isolates that exhibited the ESBL, colistin and carbapenem resistant phenotypes and detect any other AMR genotypic traits that can hitch-hike with these phenotypes by our WGS analysis. We observed both farm-scale difference where more MDR was detected in MSFs and XDR in the MSF NC cohort, and we also observed the zoonotic spill-over effect in the SSF from P to C to NC. Whilst limitations of the study include skewed data due to the antibiotic selection used to obtain isolates from the raw fecal samples and that pig samples were pooled whereas human samples were not. Additionally our study was limited to *E. coli* isolates therefore follow-up studies could be warranted looking at the resistome in all these groups by metagenomic sequencing enabling a broader picture.

This study presents a snap shot of resistant isolates that contribute to ESBL and colistin-resistant phenotypes as well as ARGs that can hitch-hike with these AMR traits. Our AMR surveillance data highlights the AMR/AMU problem, whilst publications based on questionnaire and interviews revealed that farmers, human and animal health professionals and the general public have little awareness of the problem of AMR or knowledge to counter-act the AMR effects (Sommanustweechai et al., 2018; Ström et al., 2018). Especially as it is well known that the down-stream effects of AMR have several stake-holders (Cars et al., 2011; Cantas et al., 2013; Dyar et al., 2018). It is important to state that attitudes are changing and the effects of AMR concerns, for instance Thailand has a National Action Plan on Antimicrobial Resistance endorsed by the Thai Cabinet in 2016 to reduce antibiotic consumption by 30% in veterinary medicine by 2021 (Sommanustweechai et al., 2018). Our and other’s data provide additional evidence to policymakers in the SE Asia region to highlight the importance of meeting AMU reduction goals and continue with AMR surveillance of farms to develop future AMU guidelines as well as gain a more general understanding of zoonotic transmission of AMR. We demonstrate a strong colistin AMR reservoir among pigs on small-scale farms which is likely maintained by colistin use. We can also see clear signs of zoonotic transmission/spill-over from small-scale farm pigs to contact humans to non-livestock contacts both genotypically (colistin ARG prevalence and abundance) and phenotypically (colistin MIC values). Therefore, we argue that AMU of colistin in animals should be prohibited due to previous colistin AMR reporting and further evidence we have generated (Laxminarayan et al., 2013; Carmo et al., 2018; Magnusson et al., 2019).

From our work we demonstrate the importance of surveillance to detect AMR in LMICs to highlight potential AMR risks as data from these countries are often scarce (Van Boeckel et al., 2019). Also, AMR global transfer events do occur—*bla_NDM–1_* and *mcr* resistance genes are prime examples, where their extenence on mobile genetic elements with other resistance can confer multiple-drug resistance that can easily be shared by a multitude of Gram-negative bacteria. Currently, we present the data of ARGs and known chromosomal mutations that are present in our isolate collection from the present-day versions of the databases available. However, on the discovery of novel ARGs or chromosomal mutations we encourage fellow researchers to use our data to see if these novel ARGs or mutations are also present in our study conducted in 2018. Furthermore, we would like to highlight the importance of combining interventions to reduce AMU with surveillance to reduce the resistance to antibiotic drugs such as colistin, ciprofloxacin and gentamicin and maintain the potency of drugs such as meropenem. This could be done by utilizing phenotypic AST microbroth plate testing and genotypic WGS analysis like our study or by traditional techniques such as AST disc diffusion testing and PCR used in other studies in Thailand (Lugsomya et al., 2018; Ström et al., 2018; Wangchinda et al., 2018; Ström Hallenberg et al., 2019) or even using high-throughput technologies such as AST by microfluidics or metagenomics. Our results indicate that farm-host group differences are a result of mobile genetic elements containing ARGs that are transferred from transient *E. coli* strains to endogenous *E. coli* strains by horizontal gene transfer.

## Conclusion

Using our One Health multi-factorial model to compare *E. coli* isolates from livestock, contact humans and non-contact humans on small-scale and medium-scale Thai pig farms we demonstrated: (i) prolific multi-drug resistance that was more prevelent on medium-scale farms and (ii) likely zoonotic transmission of colistin resistance in small-scale farms as there was a dissemination gradient from pigs to handlers to non-livestock contacts. We highly recommend reducing the antimicrobial use in animals’ feeds and medications, especially the last resort drug colistin.

## Data Availability Statement

Raw sequence data can be obtained from the European Nucleotide Archive (ENA) under the project accession number PRJEB38313. All sequence data from computation workflow is compiled in Supplementary File 1, virulence finder unique results in Supplementary File 2 and genomic difference output is provided in Supplementary File 3. All scripts and Python code and metadata can be provided on request.

## Ethics Statement

This study was performed in accordance to the Helsinki declaration for the human subjects and the EU Directive 2010/63/EU for animal experiments; the protocol involving human participants and animals was approved by the Khon Kaen University Ethics Committee (Project ID: HE612268 and 0514.1.75/66, respectively). Informed consent for each human subject was obtained after an explanation of the experimental procedures. The patients/participants provided their written informed consent to participate in this study. Pig samples were collected at the farm with the farm owners permission of the pig herd. Written informed consent was obtained from the owners for the participation of their animals in this study.

## Author Contributions

RH, TL, UM, MS, and JDJ designed the study. KL, JJ, SA, and MS collected the original samples. RH, TL, and KL did the Laboratory work. RH analyzed the data and prepared figures. RH and JDJ interpreted the data. RH wrote the manuscript with critical revisions by JDJ. All authors revised and approved the final manuscript.

## Conflict of Interest

The authors declare that the research was conducted in the absence of any commercial or financial relationships that could be construed as a potential conflict of interest.
