# Supplementary figures and images for: Exploring the Antibiotic Resistance Burden in Livestock, Livestock Handlers and Their Non-Livestock Handling Contacts: A One Health Perspective
Source: Front Microbiol. 2021 Apr 20;12:651461. doi: 10.3389/fmicb.2021.651461 (PMC8093850; doi:10.3389/fmicb.2021.651461)

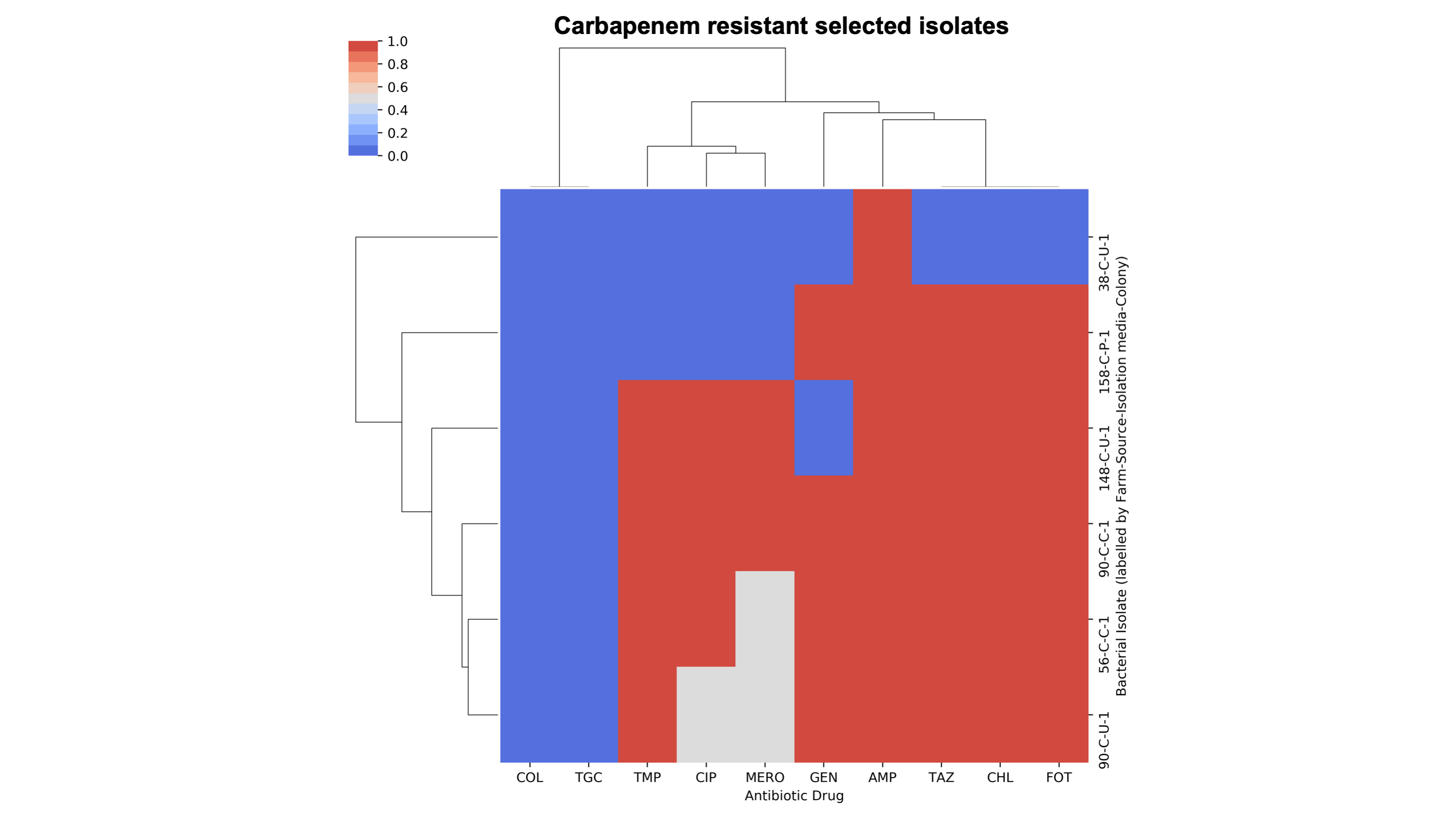

Supplement: Supplementary file 1 [file Image_1.PNG]

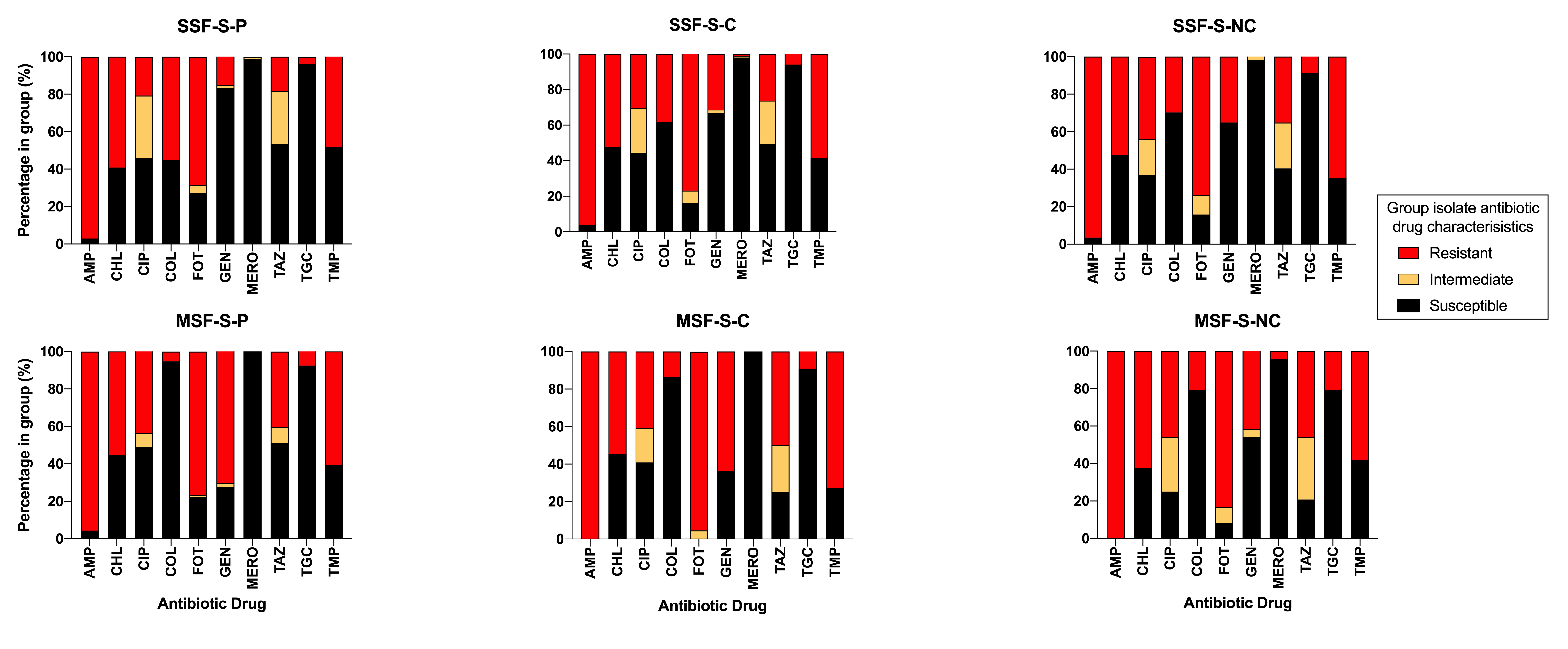

Supplement: Supplementary file 2 [file Image_2.PNG]

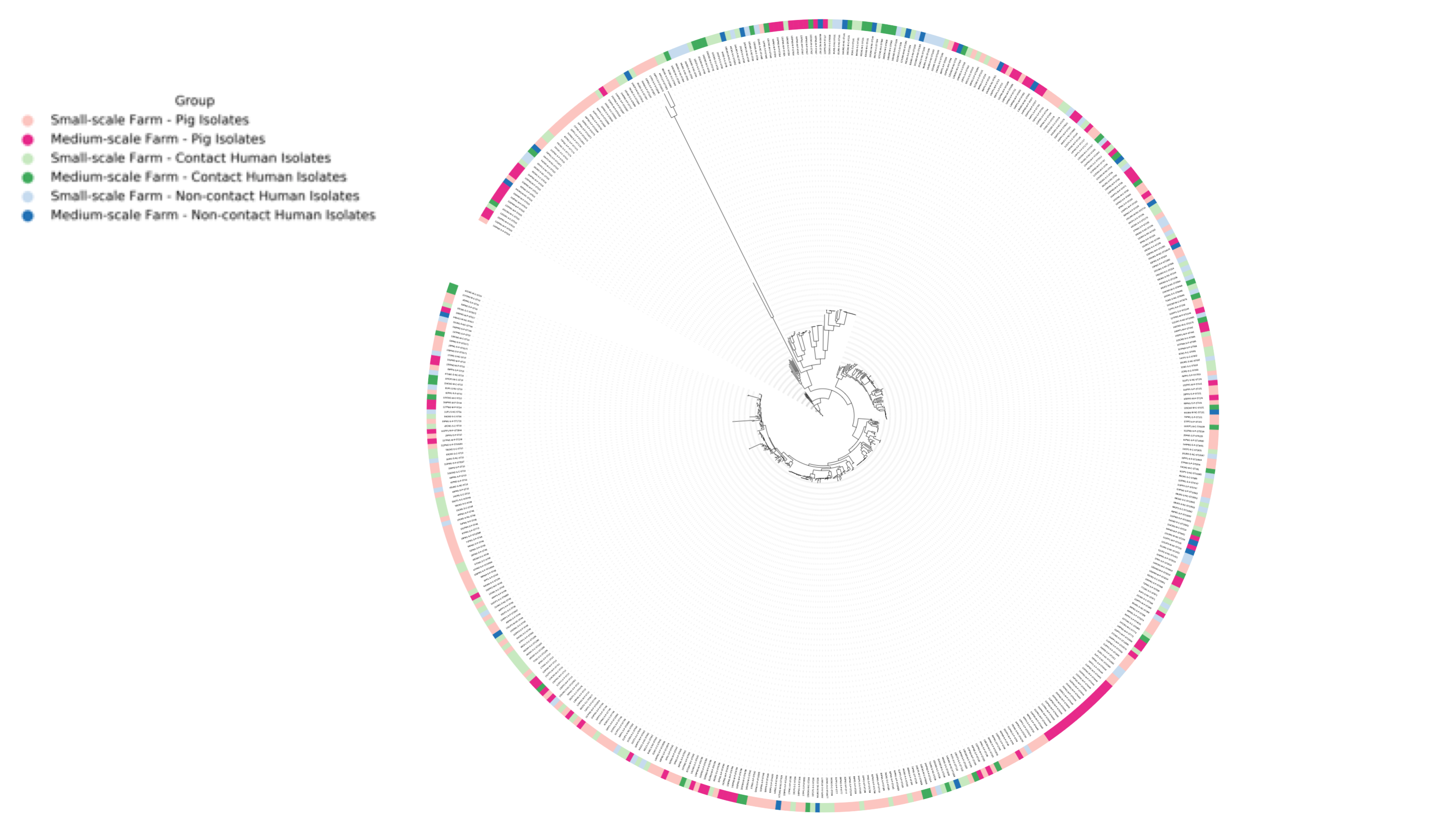

Supplement: Supplementary file 3 [file Image_3.PNG]
